# Starting from hopelessness – grand challenges, sustainable development goals, and responsible research and innovation under urgency and uncertainty

**DOI:** 10.1080/23299460.2025.2497609

**Published:** 2025-05-22

**Authors:** Sophia Efstathiou, Mrudhula Koshy, Avijit Vinayak Pandit

**Affiliations:** a Department of Philosophy and Religious Studies, Norwegian University of Science and Technology, Trondheim, Norway; b Department of Philosophy, The American College of Greece, Athens, Greece; c Department of Architecture and Planning, Norwegian University of Science and Technology, Trondheim, Norway; d Department of Industrial Ecology, Norwegian University of Science and Technology, Trondheim, Norway

**Keywords:** Uncertainty, contingency, planning, Taoism, Buddhism, improvisation

## Abstract

International governance has responded to global challenges like climate change, poverty or health through the UN Sustainable Development Goals, or Grand Challenges for research. This paper provocatively proposes that, besides goal-setting, responding to urgent societal challenges can start from hopelessness. Starting from hopelessness, attends to, and accepts a given situation, and harvests its potentials. Building on Jullien's work on Taoist approaches to effective action and Bovens's definition of hope, we define starting from hopelessness as (a) desiring an outcome, (b) believing it is possible, though uncertain, and, instead of imagining things otherwise, (c) accepting the grim situation one is in, harvesting what potential it offers without pre-set plans. The principles of acceptance, humility and togetherness help guide hopeless action. We illustrate divergent crisis responses, starting from goals or from hopelessness, through cases in Orissa and Wayanad, India. We further explore what ‘hopeless planning' and ‘hopeless research and innovation’ may involve.

## Introduction

Armed conflict and forced displacement, climate catastrophe, food insecurity, energy poverty, global health, social inequality: the sheer scale of ‘grand’ or ‘global’ challenges that are urgent to address collectively is growing. But how can we best address such complex, interconnected challenges in an age of ‘polycrisis’ (cf. Søgaard Jørgensen et al. 2024; Zeitlin, Nicoli, and Laffan 2019)?

Humanity approached this millennium with optimism: in the early 2000s, world leaders set some visionary, millennial ‘resolutions’ in the form of the Millennium Development Goals (MDGs). Several issues were considered, including extreme poverty, universal education, hunger and disease, which are persistent and deeply rooted challenges. Yet, the dissolution of the Soviet Union and the expansion of capitalist markets into China had created a newfound sense of unity, a ‘global minus’, as Bruno Latour called this less complex, unified whole (2018). These types of ‘grand challenges’, established as important targets for research by philanthrocapitalist actors like the Bill and Melinda Gates Foundation, and through public funding schemes (Efstathiou 2016), are arguably leading to a ‘sportification’ of research (Kaldewey 2018), whereby scientists relentlessly compete in targeting such well-funded areas.

Today’s illustration of this ‘go-get-them’ spirit is the set of 17 United Nations (UN) Sustainable Development Goals (SDGs) formulated during the UN Sustainable Development Conference in Rio de Janeiro in 2012. Adopted by the UN in 2015, the SDGs are currently supported by international research and governance (Sachs 2012; see the issue by Stevens and Kanie 2016). Like the MDGs, the SDGs are a democratic feat: they capture a collectively desirable future for humanity. Framed around positive concepts like ‘no hunger’ or ‘zero poverty’, the SDGs add value for enterprising economies, providing an overall sense of meaning, direction and, importantly, *hope*.

This leads to our central research question: Are there other, perhaps more efficacious, ways to respond to grand challenges besides setting goals? Our suggested approach lies in exploring the paradoxical concept of addressing grand challenges *starting from hopelessness*. Our aim is not to suppress hope but to pause and attend to another important, though perhaps ‘backwards’, feeling: hopelessness. We highlight how going forward may involve working on the sidelines of – or even in opposition to  – accepted goals, including moving ‘sideways’ or ‘backwards’ (García Zarranz 2020; Stockton 2009). Recognising these movements as vital for addressing urgent grand challenges we develop an approach that embraces them: what we dub *starting from hopelessness*.

We define *starting from hopelessness* as:

- having a desire for an outcome,
- believing that this outcome is possible, but
- accepting the grim situation that one is in and harvesting any potential this situation may offer, without pre-set plans.

We further articulate *hopeless action* through three practical principles:

- Acceptance: accepting the situation that one is in, appreciating whatever it offers;
- Humility: embracing failure and uncertainty as integral parts of creative action;
- Togetherness: staying vulnerable and working together with others, human and nonhuman.

We hypothesise that addressing the climate crisis or other ‘grand challenges’ by starting from hopelessness can facilitate real-time adaptation, frugal innovation and constantly evolving collaboration. We further suggest that starting from hopelessness can, in some cases, work better than setting goals, harvesting opportunities that would have been missed if goals were pre-set, and goal-achievement plans followed.

The paper's first main section introduces its inspiration: Francois Jullien’s analysis of different approaches to efficacy within Indo-European and Chinese, Taoist cultural traditions ([2005] 2012) (Section ‘Two approaches to efficacy: planning to change versus accepting a situation’). The next section adopts the definition developed by philosopher and decision theorist Luc Bovens (1999) to articulate our concept of starting from hopelessness (Section ‘Defining an alternative to pre-planned action: starting from hopelessness’). Two real-life cases of crisis response in India are used to illustrate goal-driven, top-down planning, versus the improvised, decentralised responses (Koshy 2022; Koshy, Aranya, and Refstie 2022; Sainath 1996), which we analyse as starting from hopelessness (Section ‘Case-studies from India: limitations of rigid goal-setting and the potentials of embracing a grim situation’). This approach is further explored by contemplating what ‘hopeless planning’ and ‘hopeless research and innovation’ might involve while building on established concepts in these fields (Section ‘Starting from hopelessness in Planning and Responsible Research and Innovation (RRI)’). We conclude by reflecting on the hopelessness of starting from hopelessness and inviting.

## Two approaches to efficacy: planning to change versus accepting a situation

Francois Jullien’s book *Conférence sur efficacité* distinguishes between two approaches to efficacy, or ‘what works’ ([2005] 2012). The first is setting one’s goal, developing a plan to achieve this goal and implementing the plan (Jullien [2005] 2012, 22). The second is being in a situation and harvesting the potential of this situation (Jullien [2005] 2012, 40–41). Jullien attributes the first approach to Indo-European cultural traditions and the second to Taoist and Chinese traditions.

Within Indo-European traditions, effective action is chiefly conceptualised as applying a ‘theory’, or ‘plan’ to ‘practice’. As Jullien observes, these traditions prominently distinguish between ‘theory’ and ‘practice’ ([2005] 2012, 22–23). God, nature, or chance are imagined as acting apart from humans – different from, but in the image of humans – as anthropomorphised gods. Further, many Indo-European cultures draw a distinction between heaven and earth, separating where gods and mortals reside. This parallels how theory is imagined to be ‘up in the sky’, while practice happens ‘on the ground’ (Jullien [2005] 2012, 50–51). A characteristic product of Indo-European culture is epic poetry, featuring heroes celebrated for facing and overcoming great obstacles (Jullien [2005] 2012, 69).

Effective action is understood differently in Taoist and Chinese thinking (Jullien [2005] 2012). Reality is here conceived as a continuous process of imminent transformation. Even though there is no word for ‘nature’, there is a term for ‘sky’ as a rhythmic and continuous process, and there is also a term for ‘sky and earth’ as a polarity through which a relation is born (Jullien [2005] 2012, 63–64). Key concepts in Chinese thinking include ‘the field’, that is, a given ‘situation’, and ‘shi’, which stands for the propensity, or the tendency, of things (Jullien [2005] 2012, 40). Humans, as part of nature, thrive when they align their actions with the potential of their situation and surroundings. In this tradition, emphasis is placed on process and on orienting oneself in a transforming situation so as to avoid meeting obstacles.

The classic Chinese text *Tao Te Ching*, dated to the fourth century BCE and attributed to the philosopher Lao Tzu, emphasises adaptability and flexibility as key virtues for harmonising with life’s natural rhythms. It encourages avoiding rigid plans, being open to change and unexpected outcomes.

Working ruins, grasping loses. And so, the Wise Person: does not work, so does not ruin, does not grasp, so does not lose. (Tzu 2007, Chapter 9)

This can be interpreted as a call to embrace adaptability in one's approach towards challenges. From this perspective, if one is too focused on results they may lose sight of what matters. Lao Tzu notes:

The soft overcomes the hard. The gentle overcomes the rigid. (Tzu 2007, Chapter 36)

Value is placed on a gentle and flexible approach, avoiding forceful, or aggressive action.

Following Jullien, these divergent ways of thinking are reflected in Western and Chinese traditions’ writings on war strategy. As war and armed conflict are, indeed, a form of crisis, the passages cited below illustrate these differing approaches towards crisis.

In the words of Carl von Clausewitz, a prominent strategist who fought in the Napoleonic wars as part of the Prussian army:

Activity in war is movement in a resistant medium. Just as a man in water is unable to perform with ease and regularity the most natural and simplest movement, that of walking, so in war, with ordinary powers, one cannot keep even the line of mediocrity.

This is the reason that the correct theorist is like a swimming master, who teaches on dry land movements which are required in the water, which must appear grotesque and ludicrous to those who forget about the water. This is also why theorists, who have never plunged in themselves, or who cannot deduce any generalities from their experience, are unpractical and even absurd, because they only teach what everyone knows—how to walk. (Von Clausewitz 2003, Book 1 chapter 7)

The excerpt illustrates the distinction between theory and practice: a good teacher teaches from their experiences, without depending only on theoretical knowledge. Still, a good general sticks to their plan: they accept the challenges, friction, and gaps between their plan and reality, and adapt accordingly (Jullien [2005] 2012, 33).

We could also interpret the words of the Chinese theorist of war Sun Tzu as regarding crises. Written in the 6th century BCE, his book, *The Art of War* advises: ‘to fight and conquer in all your battles is not supreme excellence: supreme excellence consists in breaking the enemy’s resistance without fighting’ (Tzu 1963, Chapter III: paragraph 2). Emphasis is placed on adapting to situations and demands without coming to conflict.

Military tactics are like unto water; for water in its natural course runs away from high places and hastens downwards.

So, in war, the way is to avoid what is strong, and to strike at what is weak. (Tzu 1963, Chapter VI: paragraphs 29, 30)

Instead of the friction described by von Clausewitz, this approach values ease. Patience becomes a skill for harvesting the potential of a situation:

Disciplined and calm, to await the appearance of disorder and hubbub amongst the enemy: this is the art of retaining self-possession. (Tzu 1963, Chapter VII: paragraph 30)

According to this account, a good strategist attends to evolving situations and avoids struggle. Contrasting the Indo-European ideal of a hero, a good general in Taoist thinking may never overcome any obstacles because they skilfully avoided them (Jullien [2005] 2012, 42–43).

This approach to effective action relies on embracing change, and cultivating an openness to harvest the potential of an evolving situation instead of following a pre-formed plan. This we dub, in contrast to rigid goal-setting*, starting from hopelessness.* We present this concept below.

## Defining an alternative to pre-planned action: starting from hopelessness

Hope is difficult to give up, especially in the face of looming climate catastrophes and impending societal, political, and humanitarian crises. It seems almost criminal to suggest that we embrace hopelessness. Would this not lead to a paralysis of action?

Hopelessness is analysed here not as despair but as a deflation of hope: a hope-less surrender. This amounts to a deep acknowledgement of limits, uncertainty and transience for all solutions. We suggest that starting from a place of emptiness is starting from a place of openness, thus making room to appreciate what a situation and others (human and nonhuman) have to say and to offer. Perhaps hopelessness is paralysing, but a temporary pause can catalyse the change that inertia and routine hinder.

To articulate this proposal, we build on the work of philosopher and decision theorist Luc Bovens on hope and that of Buddhist nun Pema Chödron on existential uncertainty. Several contemporary philosophers, especially of the ‘Continental’ philosophical tradition, have written on affects like despair, anguish, or anxiety. To offer some examples, Søren Kierkegaard discusses despair as a failure to achieve one’s ‘higher self’ in *The Sickness unto Death*; Jean-Paul Sartre discusses the anguish of a free-willed existence in *Being and Nothingness*; Martin Heidegger analyses anxiety, stimulated when separated from being-in-the-world, and in being-towards-death, as in part revealing the possibility of authentic being. We find that, for the purposes of this analysis, the marriage of analytic with Taoist and Buddhist thinking provides a more generative starting point for formalising this discussion.

Hoping for a situation S – for example Sustainability  – is defined by Luc Bovens in his paper on hope (1999). Bovens defines *hoping* as a conjunction of the following three conditions (1999, 673–674):

1. first, having a desire for the state of affairs S,
2. second, holding a belief that S is possible, and
3. third, engaging in some mental imaging, visualisation or thinking about S happening.

All three conditions are necessary for hope, according to Bovens (1999).

To demonstrate his definition of hope, Bovens uses the example of two characters from Frank Darabond’s film, *The Shawshank Redemption.*^1^ This American movie, a blockbuster of the 1990s, tells the story of two prison inmates, Andy and Red, played by Tim Robbins and Morgan Freeman, respectively. Andy wants to escape, believes it is possible, and hatches a plan to achieve this goal. He would be the example of a person hoping. Red also wants to get out one day, and he believes that one of the committees assessing his behaviour could grant him a release, but he is not actively planning to get out. Indeed, he thinks that hoping is a waste of time and that it will only lead to disappointment.

We define ‘starting from hopelessness’ by accepting the first two conditions Bovens specifies for hope and negating the third. We consider engaging in mental imaging, visualisation or thinking about S happening as part of developing strategies for achieving goals like S. Admittedly, hoping for S and setting S as one’s goal are different actions. If one sets S as a goal, and given one is realistic about their goals this would imply hoping for S, and indeed, expecting S to happen. Hoping for S can thus be thought of as a starting point in making S-achievement into one’s goal, with thinking about and visualising S being key in charting out ways to achieve this goal.

Starting from hopelessness, instead of hope, as we define it, involves:

- having a desire for an outcome S,
- holding a belief that this outcome is possible, while recognising it is uncertain, but
- accepting the situation one is in, without any mental imaging, or planning for it to be otherwise.

Note that starting from hopelessness does not imply dwelling in despair – even though humanity has every reason to despair. Human activities have already caused a global warming of 1.0°C, which is likely to reach 1.5°C, even in the low emissions scenarios tested by the Intergovernmental Panel on Climate Change (IPCC 2021, 56). Exceeding 1.5°C will have catastrophic consequences, including more frequent and severe heatwaves, droughts, floods, wildfires, and sea level rise, impacting millions of people and causing irreparable damage to ecosystems and biodiversity (IPCC 2018). The decade of the 2020s is crucial to alter this prospect. Yet, following a small COVID-19-induced dip, carbon emissions keep increasing.

One might thus justifiably lose hope, when faced with humanity’s collective inertia and inability to mitigate climate change. However, our ground for starting from hopelessness is not that catastrophe is staring us in the face. Rather, hopelessness offers a starting point that allows us to embrace what is happening, and accept our vulnerability, instead of denying the gravity of the situation and shifting our attention to what we wish was happening. In this regard, hopelessness creates space for accepting the complexity of a given situation, without letting one’s imagination fill in the gaps. Though this may strike the optimistic planner as a grim starting point, grimness, grief and pain are important aspects of reality.

Another way to appreciate hopelessness is through the words of Buddhist nun Pema Chödron (1997). Starting from hopelessness resonates with what Chödron calls ‘groundlessness’:

When things fall apart and we’re on the verge of we-know-not-what, the test of each of us is to stay on that brink and not concretise. The spiritual journey is not about heaven and finally getting to a place that’s really swell. In fact, that way of looking at things is what keeps us miserable. Thinking we can find some lasting pleasure and avoid pain is what Buddhism calls samsara, a hopeless cycle that goes round and round endlessly and causes us to suffer greatly. (1997, 19)

Here, Chödron addresses the actions of individual beings, yet the same line of reasoning can be extended to collective actions, such as economic or environmental policy: in fact, through the MDGs or the SDGs policy can perpetuate a ‘samsara’ of goal-setting. Thus, while setting a goal may appear as a ‘heaven’ that will liberate us from suffering, it can shift focus away from addressing the inevitable challenges of life.

The very first noble truth of the Buddha points out that suffering is inevitable for human beings as long as we believe that things last -that they don’t disintegrate, that they can be counted on to satisfy our hunger for security. From this point of view, the only time we ever know what’s really going on is when the rug’s been pulled out and we can’t find anywhere to land. We use these situations either to wake ourselves up or to put ourselves to sleep. Right now –in the very instant of groundlessness– is the seed of taking care of those who need our care and of discovering our goodness. (1997)

The rug is pulled, and the fire in our house is burning (Thunberg 2019). One may desire and believe that things can get better, but embracing hopelessness allows us to fully attend to reality in all its aspects. One may judge a situation as good or bad, but it is hard to know what is what in a process of change.

Responding to an urgent challenge starting from hopelessness can be summarised through three practical principles:

- **Acceptance:** Starting from hopelessness implies accepting what a situation has to offer, and building on available resources. Instead of investing energy to criticise others or oneself, or to ‘correct’ the situation, one puts energy into recognising, activating and working with existing resources.
- **Humility:** Starting from hopelessness involves accepting failure. There is no shame in failing nor in one’s efforts falling short. Instead of pride and attachment to one’s ideas or plans, starting from hopelessness involves cultivating humility and embracing imperfection, limits and failure as part of transformation.
- **Togetherness:** Starting from hopelessness embraces vulnerability and the need for togetherness. Vulnerability invites collaboration with others – human or nonhuman  – recognising our interrelations and need for each other. A solution to a problem emerges from a combination of approaches and from people and other living beings or things working together to move a situation forward.

Flexibility and creativity underline these responses, which adapt dynamically to evolving situations and relations. Grounded in the principles of acceptance, humility, and togetherness, starting from hopelessness embraces improvisation, frugal and piecemeal innovation, and ad hoc, or devolved modes of organisation, as viable ways to respond to crisis.

Next, we illustrate these principles through real-life examples.

## Case-studies from India: limitations of rigid goal-setting and the potentials of embracing a grim situation

We consider two types of response to a crisis. One can be described as goal-setting and planning inspired by a strong vision and implemented via a top-down leadership structure. In contrast, the other involves multiple, real-time and improvised responses from a diverse set of stakeholders. These two cases serve as ideal types in our argument, showing how each one works in a real-life situation. We do not claim that any crisis response can be neatly characterised as falling under one of these two categories of rigidly planned, versus hopeless action. Yet we propose that this distinction can help us analyse features of hierarchy and control, and of devolved action and improvisation as part of these responses.

The first example we discuss comes from the work of the Indian journalist Palagummi Sainath, a leading expert on poverty.

### Case 1. Drought in Orissa, India: where goal-achievement plans are too rigid

In his book *Everybody loves a good drought*, Sainath considers a case in India, where a planning process with inspired, but rigid, goals led to an absurd array of challenges (1996, 5–21). In the late 1970s, a network of stakeholders comprising NGOs, a private company, state banks, and national and regional governments came together to address poverty among the cattle farming community in the state of Orissa. The goal set by the government was to provide as many farmers as possible with a new ‘miracle’ cow, known for its high milk yield. These cows were brought all the way from across the country, where they lived. To feed the cows, the government also created a scheme to grow a new, high-yielding tree on local farmlands. Some villagers were forced to stop growing wheat and rice to grow the splendid fodder tree. Many stakeholders, including policymakers, NGOs, and banks, proudly claimed that they had managed to provide local employment and a long-lasting source of income for the poor farmers.

Two years later, the thousands of trees planted in the grasslands could not adapt to the local environment. Once cut for fodder, they could not grow again. The miracle cows did not adapt to the local climate either – not one extra litre of milk could be produced. The price of milk and ghee started rising dramatically. The local bull, who was resilient to the climate, was relentlessly castrated and almost driven to extinction to protect the imported cow. There were also further unintended consequences. The farmers started to migrate from the region in search of livelihood options, schools began to run empty, and the trust of the people of Orissa in the government began to diminish. Both local people and the environment suffered immensely as a result.

The overarching goal of this policy was to alleviate poverty among Orissa’s cattle farmers.

Subsidiary goals and goal-achievement plans were set with good intentions and based on what seemed to be good evidence. They were, however, implemented rigidly, and the indicators set for the implementation of the goals were too specific, ignoring how the goals were changing the farmers’ traditional livelihoods and the local economy. Instead of attending to the situation in Orissa, evidence of the success of the ‘miracle’ cow elsewhere was deemed sufficient evidence for its success in this setting. However, exporting this causal claim to the setting of Orissa did not work because of various confounding factors including the local climate and the novel plants and animals’ responses to it (cf. Cartwright and Efstathiou 2011).

The investment in a new cow and fodder tree system also created a financial lock-in, as farmers were encouraged to shift their production away from local breeds. This investment left little room for course correction once the farmers had made the shift, as farmers could not simply bring back local, climate-resilient plants and animals. Sainath attributes the failure mainly to negligence and lack of coordination (Sainath 1996, 9). But the case, as he describes it, suggests there was also an emotional attachment to the goals that made it difficult to make the necessary course corrections. Pride and resistance to accepting failure created an emotional lock-in that further prevented changes from being made (Sainath 1996, 14).

Sainath’s study of Orissa is a case of goal-achievement plans backfiring. We want to say that this was a very rigid plan  – but one might argue that it was just badly thought out. If there had been a flexible approach to planning, the plan could have worked. Perhaps the outcomes would have been different if there had been a better use of local knowledge – in alignment with principles of inclusion, reflexivity and responsiveness, which we discuss later. Indeed, goal-setting is not problematic *per se,* if there is flexibility and attention to an evolving situation.

To illustrate the importance of flexibility and adaptability, we consider another case focusing on emergency responses to floods in Wayanad, India.

### Case 2. Floods in Wayanad, India: where the potential of a grim situation is harvested

Wayanad is a peri-urban, landlocked hill district in the coastal state of Kerala, India. Primarily an agrarian economy, Wayanad is home to the largest percentage of marginalised indigenous tribal populations in Kerala, and it’s considered Kerala’s least developed district (IIA and CTP 2018). The exploitation of the land through the cultivation of commercial crops has reduced the fertility of the soil. The mountain areas are considered ecologically fragile due to excessive quarrying, while unsustainable building construction has destabilised the hilly areas, making Wayanad susceptible to landslides (Gadgil et al. 2011).

In 2018 and 2019, Kerala witnessed unprecedented floods and ensuing landslides affecting more than 5.4 million people, resulting in the loss of over 400 lives (CWC 2018), and economic losses exceeding $3.8 billion (UNDP 2018). Wayanad was one of the worst affected districts suffering a collapse of several thousand homes, and heavy damage to infrastructure, services, and land (IIA and CTP 2018). Since the last known heavy floods in Kerala were in 1924, communities and decision-makers had no personal nor institutional memory of how to deal with the catastrophic aftermath. Despite limited hands-on experience, resources and know-how, a diverse set of actors, from district officials to community volunteers, proactively took on the tasks of rescue, relief, rehabilitation, and recovery through various spontaneous and ad-hoc localised actions (Koshy, Aranya, and Refstie 2022).

The overarching desire here was to alleviate the crises created by the flooding. This resulted in partially coordinated action without pre-set goals but with multiple initiatives pursued simultaneously through improvised action. For example, a community radio service used its existing station signal to issue early warning messages (Koshy and Smith 2024). They brought district officials on air to assuage the fears of communities, as radio was one of the few available sources of news during limited access to electricity and mobile phone connectivity. Another initiative was the campaign called ‘Donate a Cow’. This was jointly coordinated by the district administration and a local NGO, inviting farmers and communities whose flocks had survived the flooding to help restore the livelihoods of those who lost their cattle during the floods (Koshy and Smith 2024). The women-led social entrepreneurship platform *Kudumbasree*, which had been set up for women from low-income communities, became crucial in mapping and assessing post-disaster community needs. Through attending to and responding to their communities’ needs, Kudumbasree members had key knowledge about the situation on the ground. They were familiar with their neighbourhoods, the condition of their settlements and the terrain, as well as the health, economic and social needs of their communities. These women’s knowledge about the situation at hand and the pre-existing organisation among them was recognised by municipal officials who included them in developing response tools, training them to document information on an app which was used for the fair distribution of relief measures.

Importantly, involving multiple actors to respond to the floods on the ground and in real-time informed planning, prevention and adaptation actions anticipating future catastrophic events. Following the two years of floods, numerous incremental institutional changes were put in place to ensure a better response for plausible future flood situations. This included training local youth as a task force for rescue operations, conducting emergency drills and training in schools, making village-level disaster management plans mandatory, and piloting risk-informed plans to better integrate short-term with long-term planning measures.

These are among several examples from Wayanad that illustrate how, in the face of a grim situation leading to an unprecedented cascade of crises, a diverse range of actors accepted and built on human and nonhuman resources available to them, tried, failed and improvised together through small actions and social learning from their own, firsthand experiences. It is important to recognise that Kerala’s socio-cultural and political tradition includes a history of collective engagement and social movements by working-class communities (Bardhan 2002; Heller 2001). These social ties and experiences, though unrelated to flooding, facilitated a bottom-up disaster response combining community resourcefulness and existing decentralised governance (Koshy 2022). Thus, several actors with varying formal and informal capacities, individually and collectively, accepted this grim situation and harvested what institutional, material, and social potential it held. They responded to this crisis with resourcefulness, while acknowledging the need to prepare for future crises (Koshy 2022).

### Reflections

These two cases illustrate contrasting approaches to responding to a crisis. The first case showcases how an externally imposed, top-down policy relying on goal-setting, planning and implementation led to lock-ins and self-defeats due to its rigidity and unintended ramifications. The second case highlights how a more flexible approach that starts by accepting a grim situation and responding to needs as they arise provides space for adequate but frugal innovation. These approaches we would refer to as, respectively, starting from rigid goal-setting and hopelessness. We summarise the main takeaways in the table below:

**Table:** 

Key characteristics	Orissa, India/goal-setting	Wayanad, India/hopelessness
Structure of decision-making and governance	Top-down decision-making, led by government and industry actors	Decentralised governance and decision-making, multiple local community leaders, diverse range of formal and informal actors
Temporality of response	Linear, future-oriented, causal understanding of effective problem-solving actions, organised and executed consecutively	Multiple, incremental, organic actions executed simultaneously through decentralised leadership
Participation in response	Policy decisions implemented without consulting local stakeholders and experts; local animals and flora seen as problems; knowledge gained in the process not shared with the community	Local actors and organisations involved in the crisis response as consultants and lay experts; local cattle seen as a resource; knowledge derived from the crisis response shared through training and policy plans involving the community
Starting point	A resistance to the characteristics of the local situation: local cattle and local plants seen as problems which the crisis response sought to correct	Responses accepted the grimness and severeness of the unfolding crisis, finding and exploring resources available to utilise in real-time
Attitude to learning	Denial of the plans’ failure; pride and attachment to original plans; reluctance to correct course	Adaptive and flexible attitude; building on community capacities, indicating humility; learning by failing and doing
Attitude to collective action	Taking and maintaining control; assuming locals know less, and local practices need to be changed; pursuing change through policy developed with an attitude of domination	Embracing vulnerability; recognising resources in whom and what is available; fostering togetherness and deciding action in modes that are decentralised

One of the main conclusions we can draw is that accepting the situation one is in matters both when planning fails, but also for improving planning practices. If officials in Orissa had mechanisms for engaging with local communities and local knowledge, starting their planning by considering the situation that local communities were in, a different approach would have been possible. Engaging with stakeholders in an inclusive and responsive manner has been specified as key to pursuing Responsible Research and Innovation (Owen, Bessant, and Heintz 2013), as discussed later.

We have proposed that starting from hopelessness is a viable way to respond to urgent grand challenges. But where does this leave policy goals and goal-setting? Are these inherently problematic?

### Policy goals: can we set goals in the face of hopelessnesss?

Working from a decision-theoretical perspective, Hansson, Edvardsson Bjömberg, and Cantwell (2016) highlight four main types of self-defeating mechanisms for goals, with several interesting subclassifications (see Figure 1 in their article, 497). First, Hansson, Edvardsson Bjömberg, and Cantwell (2016) propose that a goal can be an obstacle to its own fulfilment  – what is conceived as *self-defeating goal endorsement*. For example: ‘it is my goal to have no goal’. In this case, the very acceptance of a goal analytically defeats having it. Second, making a goal known could interfere with its obtainment – what is dubbed *self-defeating goal disclosure,* as in cases like ‘my goal is to impress you’. One has likely lost one’s ‘edge’ having announced this goal (2016, 504). Third, a goal-setting activity can impede goal achievement: what Hansson et al. call *self-defeating goal-setting* (2016). For example, ‘I will fly to the climate conference where we will set our climate goals for reducing emissions’. In this case, the activity pursued to set the goal hinders it, as reducing carbon emissions is undermined by flying – though arguably instrumentally justified (2016, 502). Last, and most relevant to our discussion is the fourth type of self-defeasance: *self-defeating goal-directed behaviour*, whereby ‘attempts to achieve a goal could bring the agent further away from the goal’. Hansson et al. characterise this as a ‘wide concept’ spanning too many types of failure to cover in their paper (2016, 498).

Our discussion has critiqued goal-directed behaviour insofar as the selective outlook goals impose stifles adaptation and innovation in real-time crisis responses. We argued that goal-setting processes and goal-achievement plans risk self-defeasance when they divert attention from the situation one is in, for example, by mistakenly assuming causal claims confirmed in other settings, as with the hoped-for impact of miracle cows on Orissa’s agriculture, or by creating financial and emotional lock-ins. Conversely, attending to a given situation involves regularly and collectively reviewing and adapting both goals and related plans to fit the particular persons, places and provisions in question. Thus, in our analysis, self-defeating goal-directed behaviour is not inevitable.

Policy goals such as the UN’s Sustainable Development Goals have been critiqued on several grounds, for example for a lack of prioritising, or for being too vague: aspirational but unmeasurable (Pongiglione 2015). An *Economist* article from 2015, soon after the SDGs were announced, claimed SDG stood for Senseless, Dreaming and Garbled (Easterly 2015a, 2015b; Reinert 2020). Still, recent research from various disciplines has called for strengthening our understanding of relational and scalar interdependencies between the SDGs pointing to a more nuanced, reflexive approach to their implementation (Sareen and Nordholm 2021; Ulbrich 2021). Further work explores how to best operationalise the SDGs, for instance, for addressing societal challenges at the local level (Reddy 2016; Reyers and Selig 2020) or to better engage businesses (Van Zanten and Van Tulder 2018).

Similarly, setting science policy goals in terms of addressing grand, or global, challenges has been critiqued as neglecting the multiple, scientific and community stakeholder perspectives that would need to be negotiated to begin to address such problems (Efstathiou 2016; Ludwig et al. 2022). Instead, dominant actors often affirm their prominence by promising to (unilaterally) solve grand challenges, e.g. through a breakthrough technology (Ludwig et al. 2022). Though identifying research as addressing grand challenges can make it easier for policy-makers and industry to support this work (Välikangas 2022), in practice, even when organised around a ‘grand challenge’, the targets that academic research aims for are ones like competitiveness, and publications (Välikangas 2022; see Felt et al. 2016).

Our proposal is that bridging the gap between top-down, or ‘grand’ goal-setting and attending to the situations in which local communities find themselves can benefit from new paradigms for understanding effective action which move away from goal-setting and planning as the only starting points. Negotiating multiple approaches and being reflexive in our understandings of SDGs and grand challenges are key, but our proposal works up to such responses from another starting point.

Starting from hopelessness builds on Taoist and Buddhist insights to propose that dynamic, ad hoc, devolved, piecemeal, found, or improvised responses may work, and indeed work better to address challenges, during crisis. We identify the principles of acceptance, humility and togetherness, as characterising such approaches, starting from hopelessness. Hopeless action is in some ways orthogonal to agreed-upon, systematic goal-achievement, but it can complement established approaches, while multiplying possibilities for effective action. Recognising and valuing responses starting from hopelessness leverages a broader range of knowledge that different actors and cultures have developed while responding to crises. Learning from such experiences and knowledge can foster better responses in the future, as we see in the case of Wayanad.

## Starting from hopelessness in Planning and Responsible Research and Innovation (RRI)

In this section, we connect Taoist and Buddhist insights on effective action with work in Planning and Responsible Research and Innovation (RRI). The reason is that starting from hopelessness resonates with existing work, and indeed, can inspire new research in these fields.

Science and planning often focus on reducing uncertainty rather than accepting it. In contrast, traditions like Buddhism and Taoism offer approaches for understanding and accepting uncertainty and impermanence while acting from a space of openness to change. There are already seeds of valuing flexibility and adaptability in these research fields. Still, we begin a new seed of thought to frame and justify why devolved organisation, and piecemeal, improvised and frugal innovations can be effective responses to uncertainty and crisis: such responses start from hopelessness.

### Embracing uncertainty: hopeless planning

The field of Planning has been progressively embracing uncertainty. Traditional ways of planning promoted rational and instrumental approaches that sought to increase certainty as a desirable goal. In contrast, recent discussions call for a more organic and contextual approach to planning by embedding uncertainty into the planning framework (Abbott 2005; Christensen 1985; Koshy, Aranya, and Refstie 2022). Incremental approaches, such as ‘muddling through’ planning problems (Lindblom 1959), and the recognition of ‘wicked problems’ (Rittel and Webber 1973) acknowledge that bounded rationalities and biases are inevitable both for individual planners and for planning institutions (Forester 1984).

Karen S. Christensen’s paper ‘Coping with uncertainty in planning’ (1985) argues that when the goal and the means of management are unknown, competencies for acting and innovating under chaos become crucial. Catastrophic events, whose probability of occurrence and degree of predictability are low, are examples of when such competencies are vital. These include situations of extreme weather, terrorism, conflict-related displacement, and economic and political crises (Koshy, Aranya, and Refstie 2022). Subsequent research deepens the conceptualisation of uncertainty in Planning calling for adaptive, relational, risk-based, and contextualised contingency planning (Brugnach et al. 2008; Koshy, Aranya, and Refstie 2022; Rauws 2017; Zandvoort et al. 2018). Such planning approaches emphasise the need to pivot and change course quickly while operating in situations of high processual and environmental uncertainty. As seen in the two contrasting examples presented earlier, rigid planning can have maladaptive outcomes, while flexible planning enables adjusting to the uncertainties of the situation.

As we see it, conceptualisations of flexible planning based on an acceptance of uncertainty and of the limits of prediction and control align with and are supported by traditional Buddhist and Taoist insights. In our understanding, acknowledging and accepting bounds, and humbly attending to a grim situation, while working collectively across social or species boundaries, are core aspects of hopeless action. This analysis plants a seed for conceptualising a range of planning initiatives as starting from hopelessness.

What we might call ‘hopeless planning’ starts by acknowledging plans and planners’ limitations, adopting a stance of humility and vulnerability not as a weakness but as a strength. Hopeless planning can add value by asking questions like:

- Are there alternatives to imposed control and forced change?
- Can situations or persons be allowed to mature into a desired state? How?
- What are the skills needed for attending to evolving situations? How can these skills be fostered?
- What are real-time, dynamic, collective, devolved or otherwise flexible approaches towards realising desired outcomes?
- Can some desired outcomes be given up without loss?

Hopeless planning need not be limited to crisis responses like the ones discussed. It can include carefully attending to and recognising the groundwork that planners need to perform to harvest the potential of situations they are in, while adapting to the resources available to them in the moment.4

### Innovating from hopelessness: hopeless research and innovation

Responsible Research and Innovation (RRI) has emerged as a further field whose perspectives resonate with starting from hopelessness. RRI, as elaborated by dominant actors in the field, developed in direct response to European policymakers’ concerns with ‘failed’ technoscience projects (European Commission 2001; Owen, von Schomberg, and Macnaghten 2021). One example is food containing genetically modified organisms (GMOs). This technoscientific project failed in Europe because it lacked societal acceptance: the impacts of GMOs were deemed scientifically uncertain and unsafe for human health and the environment. RRI aims to ensure that research and innovation are ‘responsible’: appealing to citizens’ values and concerns by aligning with consumer demands and limiting risks arising from market failures (Owen, von Schomberg, and Macnaghten 2021; Von Schomberg 2013; see Randles et al. 2022 for a richer genealogy of responsible innovation). René von Schomberg defines RRI as:

a transparent, interactive process by which societal actors and innovators become mutually responsive to each other with a view to the (ethical) acceptability, sustainability and societal desirability of the innovation process and its marketable products (in order to allow a proper embedding of scientific and technological advances in our society). (Von Schomberg 2011, 9)

Challenging the idea that the aim of innovation is market uptake and profits, RRI emphasises that ethics, sustainability and societal impacts are crucial for deeming innovation responsible. At the same time, RRI envisions approaches for doing-while-governing research and innovation processes. It distinguishes itself from risk assessment, which happens through regulatory and approval processes and from market feedback mechanisms following the creation of a product or technology.

Starting from hopelessness, by accepting what a grim situation offers, coheres with RRI’s attention to the context of technoscientific innovation. Work within technology assessment has long emphasised a real-time evaluation of technology development (Guston and Sarewitz 2002). Who are the stakeholders? What are the values at stake? How can one harvest the potential of the situation one is in? The approach dubbed ‘midstream modulation’ developed by Erik Fisher and colleagues is a landmark approach using Socratic-inspired dialogue to steer lab members in real-time to consider the ethical and environmental impacts of their work (2006). A range of approaches have been developed to integrate societal concerns into research and innovation processes (cf. Fisher et al. 2015). Yet, leaving RRI approaches unspecified allows for adapting to the needs of specific research and innovation projects (Delgado and Åm 2018). This aligns with an understanding of RRI as a learning process (Egeland, Forsberg, and Maximova-Mentzoni 2019; Kupper et al. 2015), responding to the situation one is in.

The outward, socially aware focus that is imagined for RRI aligns with the principles of acceptance, humility and togetherness that guide actions starting from hopelessness. Especially relevant here is the articulation of RRI is in terms of the ‘AIRR’ principles of anticipation, inclusion, reflexivity and responsiveness (Owen et al. 2013; Stilgoe, Owen, and Macnaghten 2013). RRI repeats key insights from feminist philosophy of science and technology by acknowledging and inviting the perspectives of minority and marginalised knowledges into scientific knowledge-making (Doezema et al. 2019; Harding 1986, 1991). Besides the expectation to anticipate the future impacts of technologies, which speaks more of planning and control, the other three principles of inclusion, reflexivity and responsiveness align with starting from hopelessness. Including a diverse set of stakeholders in the innovation process, starts from humility and fosters togetherness. Promoting reflexivity among research and innovation teams and responding to the inputs of societal actors as part of the innovation process similarly fosters acceptance of a given situation, humility and togetherness. RRI’s emphasis on inclusion and responsiveness further resonates with already discussed contingency approaches to planning, by fostering relational understandings of uncertainty. Both RRI and recent conceptualisations in planning espouse proactively embedding flexibility and adaptability into short- and long-term processes, strategies, decisions, and actions (Koshy, Aranya, and Refstie 2022). Furthermore, anticipatory perspectives in planning and RRI though aspiring perhaps to control, still, explicitly consider the temporal dimension, i.e. short-term vs long-term, as well as the desired pace and tempo of responding to societal needs (Koshy 2022; Steen 2021). RRI is envisioned as considering not only the quantitatively measurable ‘hard’ impacts of technology on health, environment or safety, but innovation’s qualitative, ‘soft’, societal or moral impacts (Swierstra 2015; Swierstra and Te Molder 2012). This includes how technology can compromise or foster different values (Van den Hoven 2007; cf. Winner 1980) – processes that are difficult to anticipate, let alone directly reroute.

RRI does, in some ways, aspire to control research and innovation and steer it in a ‘responsible’ direction. Though focusing on desired technologies, RRI enables work in real-time, looking for and finding resources and capacities afforded by a situation. Still, it acknowledges that such work needs to involve multiple actors, and that it is complex. Thus, as we interpret it, RRI stands to gain from espousing starting from hopelessness among its concepts. The principles of acceptance, humility and togetherness are open-ended, invite engagement with human (and more-than-human) stakeholders and support relational and contextual understandings of how to address societal challenges through research and innovation.

In this context, starting from hopelessness in RRI would embrace frugal and piecemeal innovation. The concept of frugal innovation was initially developed to refer to products and services created for resource-scarce contexts, but, among other applications, it has sparked interest regarding its value for sustainability (Hossain 2018; Weyrauch and Herstatt 2017). Frugal innovation can also thus be thought of as hopeless innovation, as it means developing cost-effective, low-threshold, innovative solutions that are both accessible and sustainable, using existing resources.

Another relevant concept is Marc Steen’s proposal for ‘slow innovation’ (2021). Steen (2021) argues that following the AIRR principles slows innovation down, though it creates value and enhances the quality of innovation. Steen sees slow innovation as a mark of quality, which should be celebrated as particular to European innovation (2021). From the perspective of starting from hopelessness, we share Steen’s insight that patience is needed for effective action. But time is also a resource, which may be scarce, and not afforded in given situations. In this, hopeless, spirit competition regarding best form is pointless.

What we might call ‘hopeless research and innovation’ thus considers questions including:

- Are research and innovation needed, or could there already be a working solution to this issue?
- What are the minimal resources needed and available to use in this research or innovation situation?
- Can research and innovation goals develop dynamically with evolving affordances and needs?
- Irrespective of credentials, who can act as responsible researchers and innovators in this situation?
- Are there negative actions, or non-doings that could support RRI?
- Can research and innovation be institutionally decentralised, ad hoc and still effective in addressing societal challenges?

To start from hopelessness involves appreciating and responding to the constraints and potentials of a situation at hand, frugally and sustainably. Sometimes, time for slow innovation will be afforded, and sometimes not. Hopeless research and innovation would accept with humility the bounds set by the context wherein it operates while seeking togetherness. It would embrace failures and uncertainty as part of the research process. It would acknowledge its own social, material, more-than-human, environmental and societal impacts as inescapable realities. It would navigate, like water, around/with or through them, as companions – whether they show up as resources, or as obstacles.

## Conclusion: hopeless hopelessness

This paper has argued that hopelessness offers a viable starting point for addressing grand and urgent challenges. Inspired by Taoist and Buddhist thinking about what effective action is, the paper opens a conceptual space for new modes of responding to crisis. It is an invitation to appreciate that accepting and attending to the grim situation one is in can be a generative starting point for action. Instead of continuously acting to fulfil pre-set goal-achievement plans, one can pause and embrace obstacles and limits as they evolve, and as opportunities.

Uncertainty, vulnerability and groundlessness are aspects of reality, especially regarding grand societal challenges like the currently unfolding climate catastrophe. Goals, including emissions targets, are often set with the pretence that tomorrow will resemble today. However, we live in an era where the climate is increasingly uncertain and likely to bring drastic changes, such as extreme weather events, storms, droughts, and floods. We set our goals today with the hope of stability. However, the processes we establish to achieve these goals are not equipped to handle the rapid and unpredictable changes we are already experiencing. For example, the goal of reducing emissions by 50% by 2050, often referenced in international climate agreements like the Paris Agreement, is relatively rigid and it does not fully account for the rapid changes and unforeseen challenges that will arise before 2050.

This does not imply we should dwell in despair. But we cannot blindly hope, either. To effectively manage the range of uncertainties we are facing, we need to develop flexible and adaptive approaches. Starting from hopelessness involves letting go of expectations while accepting and building on what resources are available, embracing failure without shame, staying vulnerable and working together. Incremental action, improvisation, frugal or piecemeal innovation and relying on each other are not second-rate solutions to turn to when plans fail. ‘Hopeless planning’ and ‘hopeless research and innovation’ recognise and cultivate skills, relationships, and values that can inspire efficacious ways to respond to crisis collectively.

Our proposal is not to drop goals and goal-setting altogether, as these approaches can help coordinate political action, and guide science governance. Achieving an agreement among 191 nations on 17 Sustainable Development Goals is a feat of sustained democratic process (see Chasek and Wagner 2016). As Jullien ([2005] 2012) also proposes, one of the key functions of ideal types and prototype concepts is to enable discussing and deciding on actions together as a community. Goals help to abstract from a situation and to orient towards collective action. They can also guide transdisciplinary research and innovation, by offering boundary concepts (Star and Griesemer 1989) that can be founded, measured and explored within and across disciplines (Efstathiou 2016) and negotiated (Ludwig et al. 2022). Still, it is important that the setting of goals is not just an exercise in political accountability and transparency but that it brings people together to connect to, to find motivation in, and to achieve the goals.

Hopelessness might paradoxically facilitate collective action in ways that hope blocks. Social psychologists Marczak et al. (2023) studied the emotional responses of people who describe themselves as concerned about climate change. They found that hopelessness is a very common response to problems associated with climate change. Importantly, for some participants, hope was seen as a concept obscuring an honest public discussion about the inevitable consequences of this challenge (Marczak et al. 2023, 31). Hope – in the form of policy visions  – may similarly operate to delegate, postpone, and mute discussion, by externalising a problem and blocking grassroots and personal attempts at solving it.

An important question is how to ensure transparency and monitor accountability, without pre-set goals and plans. In this case, alternative reporting and accountability structures could be developed alongside modes of devolved and decentralised social organisation. Anarchist social and organisational approaches have long been concerned with the dangers of concentrated, excess power. They explore modes of decentralised governance wherein decision-making power can be devolved to lower levels of governance and community structures (Graeber 2004). Forget about ‘The Revolution’ and go instead for ‘revolutionary acts’: ‘attempts to create autonomous communities in the face of power ’ – ones that self-organise, make their own rules or principles of operation, and re-examine them continually (Graeber 2004, 45).

This brings us to another open question. Further research is needed to deeply engage with and acknowledge Indigenous, and Taoist and Buddhist thinking in this, as in other discussions (Todd 2016; McAfee 2016). Indigenous Peoples have been active in policy circles, and some of the first to bring challenges like climate change to international attention (Kuptana 1996). In Indigenous societies, as in Buddhist teachings, compassion is the basis for action, and societies appear to operate in a state of relatedness. There is a real belief that people, objects, and the environment are connected. Humans are not considered the most important beings in the world. Instead, they become important by finding the compassion to connect with life (see for example Helander-Renvall 2010 on Sámi metaphysics).

We thus conclude by accepting the hopelessness of starting from hopelessness. Starting from hopelessness, we invite scholarly and practical collaborations to create deeper knowledge, ideas and methods for dealing with grand challenges like climate change, considering Planning, RRI, Indigenous Studies, Psychology, and many other fields.

We started this conversation by analytically contrasting hopeful goal-setting and starting from hopelessness as effective responses to crises and societal challenges. We conclude by reiterating that it is important to think of these approaches on a continuum – and, in reality, in a tangle: setting goals while improvising, hoping and accepting limits, and leading while staying vulnerable.

## Note

1 The film script was based on Stephen King’s short story ‘Rita Hayworth and Shawshank Redemption: Hope springs eternal’ in *Different Seasons*. More information on the film is available here: https://www.imdb.com/title/tt0111161/.
